# Bilateral reconstruction of the mandibular body with symphyseal preservation using a single fibula free flap: operative technique

**DOI:** 10.1186/s40463-022-00579-5

**Published:** 2022-07-28

**Authors:** Riccardo Nocini, Vittorio Favero, Luigi Chiarini, Pier Francesco Nocini

**Affiliations:** 1 Unit of Otolaryngology - Head and Neck Department, University of Verona, P.le L.A. Scuro 10, 37134 Verona, Italy; 2 Unit of Maxillo-Facial Surgery and Dentistry - Head and Neck Department, University of Verona, P.le L.A. Scuro 10, 37134 Verona, Italy; 3 Unit of Cranio-Maxillo-Facial Surgery, University of Modena and Reggio Emilia, Via del Pozzo 71, 41125 Modena, Italy

**Keywords:** Mandibular osteoradionecrosis, Fibula flap, Vessel depleted neck

## Abstract

**Background:**

Mandibular osteonecrosis may occur in 5% of the patients who undergo radiotherapy for the treatment of head and neck malignancies. Resection and microvascular reconstruction is the treatment of choice in complicated osteoradionecrosis, however multifocal presentation may complicate the management of the disease given the poor quality and limited availability of adequate recipient vessels.

**Operative technique:**

A 74-year-old man affected by multifocal severe osteoradionecrosis of the mandible underwent bilateral resection of the mandibular bodies while preserving the symphysis. The defects were reconstructed with a single fibula flap composed by two bony segments connected by a central segment, corresponding to the symphyseal region, in which the bone was dissected and removed. The anastomosis was performed on a single side of the neck. Healing was uneventful and the adopted technique allowed for a quick functional and esthetic recovery.

**Conclusion:**

The presented technique provided a safe and efficacious, although technically challenging, solution in a case presenting multifocal osteonecrosis of the jaw. The morbidity of the procedure was limited because the tissue resection and reconstruction processes were minimized.

**Graphical Abstract:**

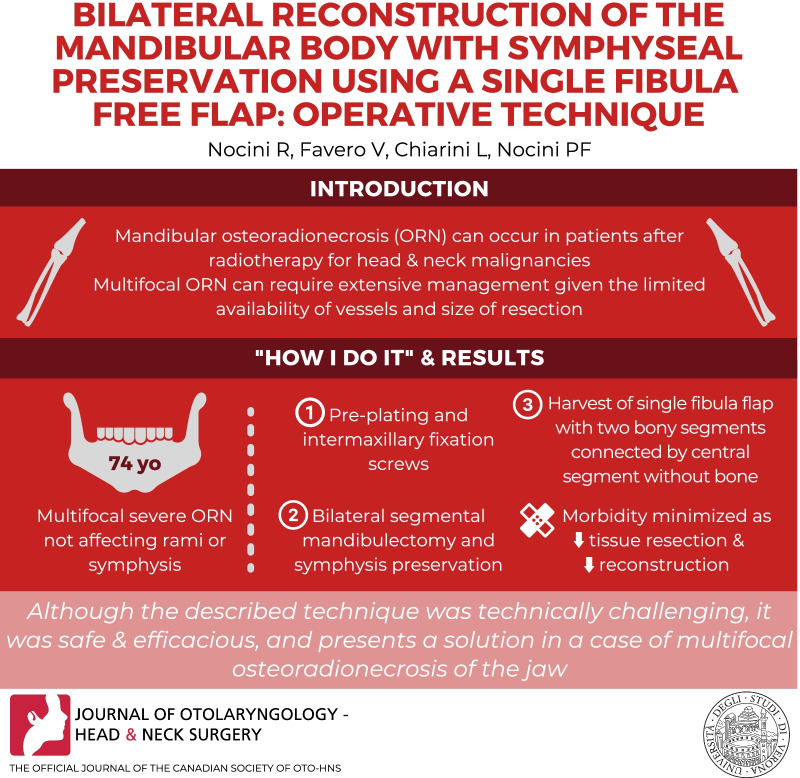

## Background

Utilized to restore form and function, Head and Neck Reconstructive Surgery has become common place in surgical practice. The use of free flaps for soft tissue reconstruction, orginally utilized for post-traumatic reconstruction, is now considered the gold standard for reconstructive surgical procedures after cancer removal [1, 2]; it is a new frontier in surgery which can be complicated by multifocal osteoradionecrosis (ORN) in patients who have undergone radiotherapy [3]. The estimated incidence of mandibular osteoradionecrosis is 5% in patients who undergo radiotherapy for malignant tumors of the head and neck [4]. How the patient is managed depends to a large extent on his/her original clinical profile. When there is diffuse or severe osteoradionecrosis of the jaw, resective surgery may be indicated [5]. It goes without saying that when microcirculation is defective, reconstructive techniques utilizing vascularized tissues represent the best option. As is well known, given its capacity to cover the defective bone and in the light of the positive role of postoperative rehabilitation, the method of choice for mandibular reconstruction is the vascularized fibula flap [6]. This flap, since its introduction by Hidalgo in 1989 [7], has gradually become the “workhorse” for the management of mandibular defects. Its peculiar features include the remarkable amount of dense cortical bone, which may account up to 25 cm in the adults, combined with a long pedicle. After its introduction into clinical practice, several surgeons adapted this flap for various needs giving birth to a number of variations of the technique in order to fit in specific defect scenarios; for instance, such variations may focus on the number and extent of skin paddles or on addressing bone height discrepancy with double barreling [8]. Cases involving osteoradionecrosis may necessitate a different type of reconstruction with respect to the ones generally used for oncologic patients as there may be extensive bilateral involvement creating a number of problems for the surgeon. The present paper describes the peculiar management of a case of severe osteoradionecrosis characterized by extensive bilateral mandibular involvement, which however spared the symphyseal region. The adopted technique yielded the advantage of using only one microsurgical flap following bilateral mandibular resection, thus reducing surgical morbidity and the greater risk of complications linked to the use of a double flap.

## Operative technique

**Fig. 1:**
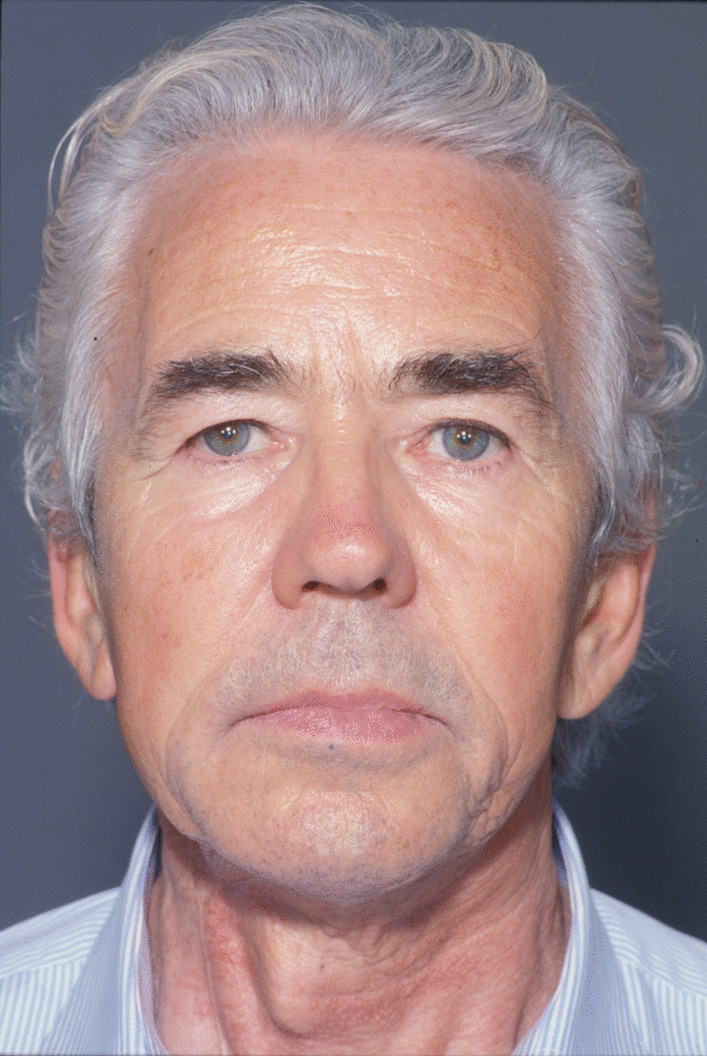
Preoperative frontal view of the patient

**Fig. 2:**
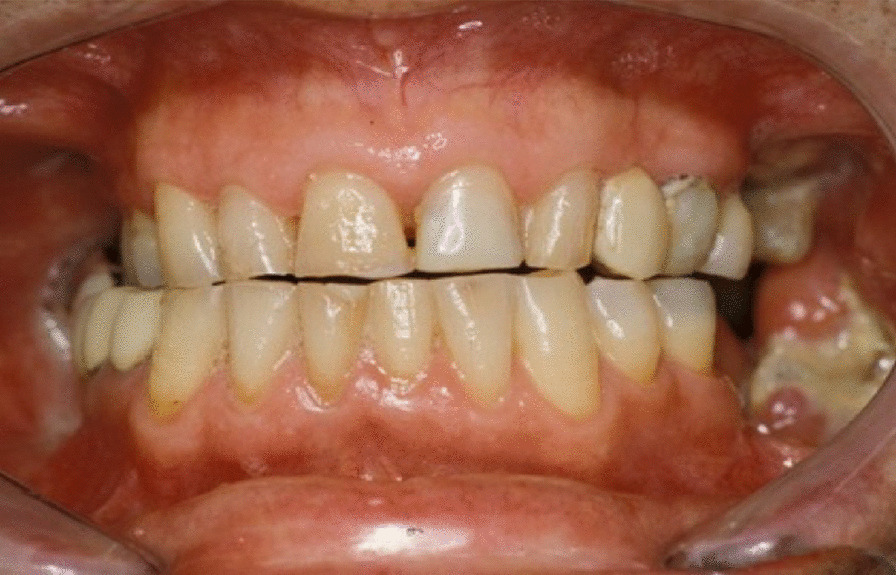
Preoperative intraoral view of the patient

**Fig. 3:**
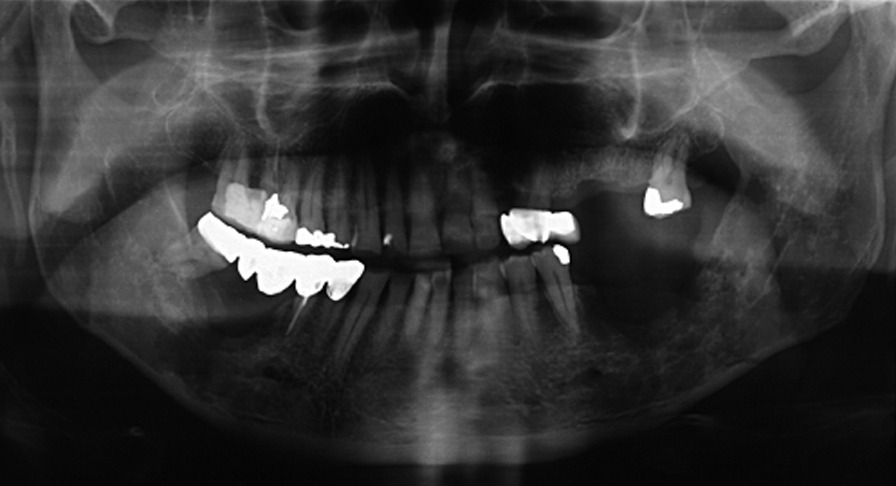
Preoperative orthopantomography

A 74-year-old male patient presenting severe ORN two years following the end of radiotherapy for a squamous cell carcinoma of the tongue came to our attention. Although the antitumoral therapy had proved successful, the patient gradually developed bone exposure on the lingual sides of the mandibular bodies. Despite surgical toilet interventions of the exposed bones and several cycles of antibiotic therapy prescribed by the patient’s previous surgeon, his clinical profile was complicated by trismus, bilateral inferior alveolar nerve paresthesia and pain. When the patient was brought to our attention, he presented signs of severe bilateral ORN of the mandibular bodies (Figs. 1,2). Neither the mandibular rami nor symphysis however presented clinical or radiologic signs of osteonecrotic involvement (Fig. 3). The therapeutic strategy that was decided upon focused on the surgical resection of both osteonecrotic epicenters while simultaneously preserving the mandibular symphysis and relative dental elements. Since the patient had undergone radiotherapy treatment, the surgical team decided to use a single vascularized fibular flap to manage the bilateral tissue loss.

**Fig. 4:**
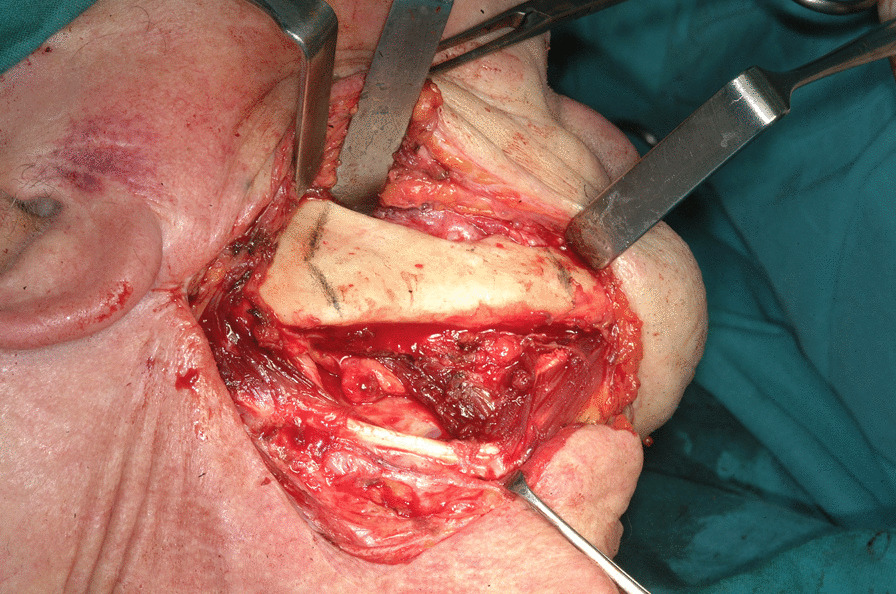
Right mandibular body, intraoperative view

**Fig. 5:**
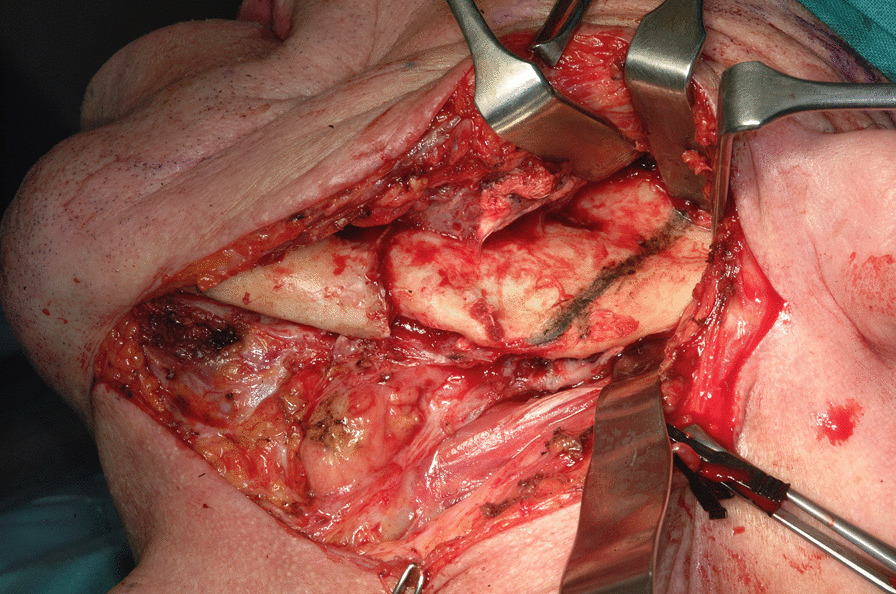
Left mandibular body with pathological fracture, intraoperative view

**Fig. 6:**
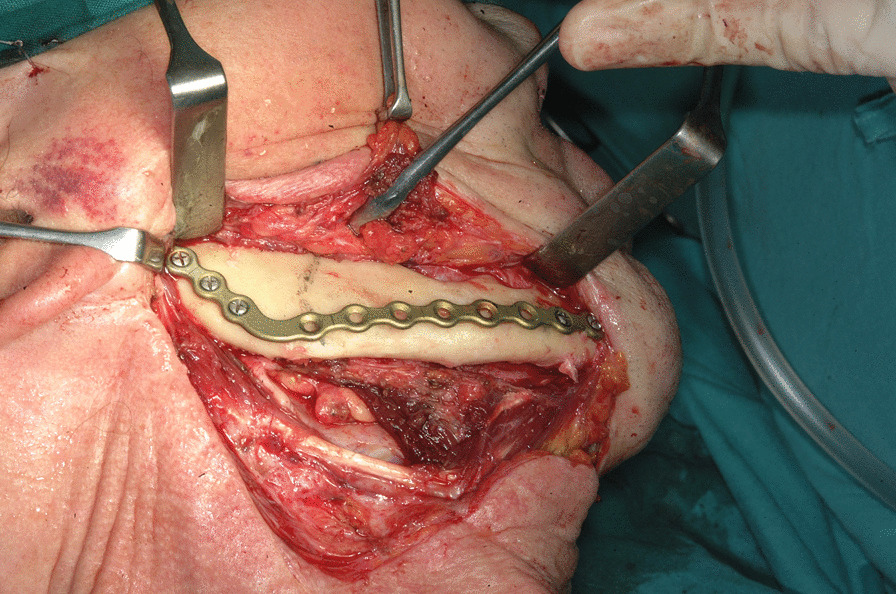
2.0 mm. reconstruction plate applied to the right mandibular body

**Fig. 7:**
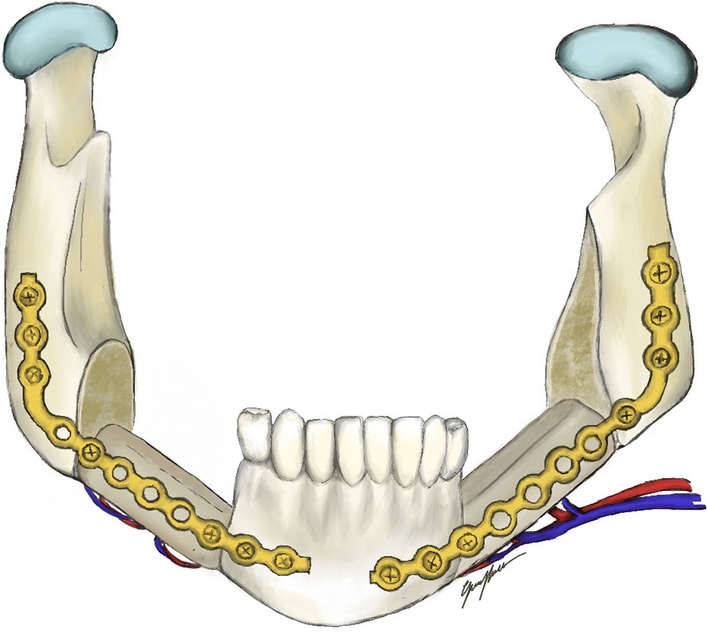
Illustration of fibula flap with central bony segment removed

**Fig. 8:**
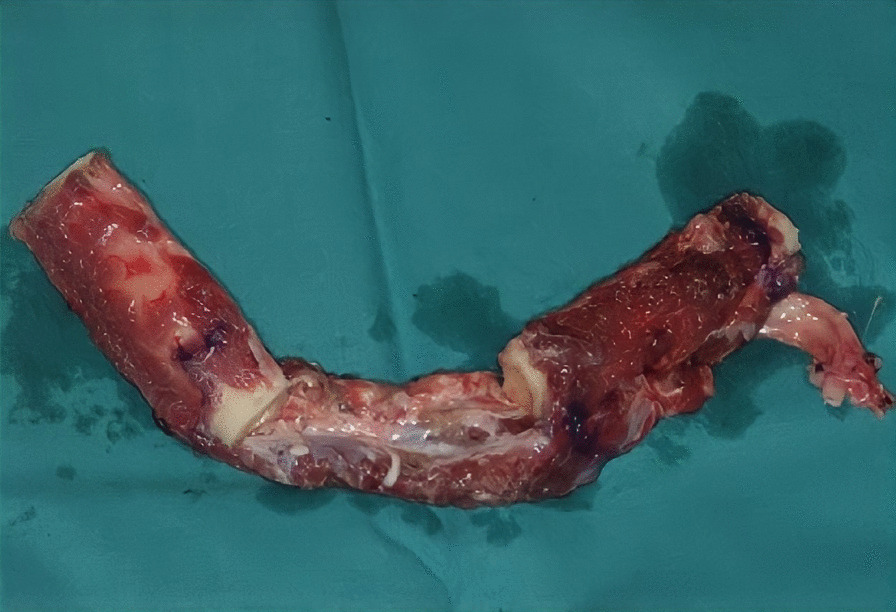
Fibula flap with central segment removed and resulting periosteal bridge

**Fig. 9:**
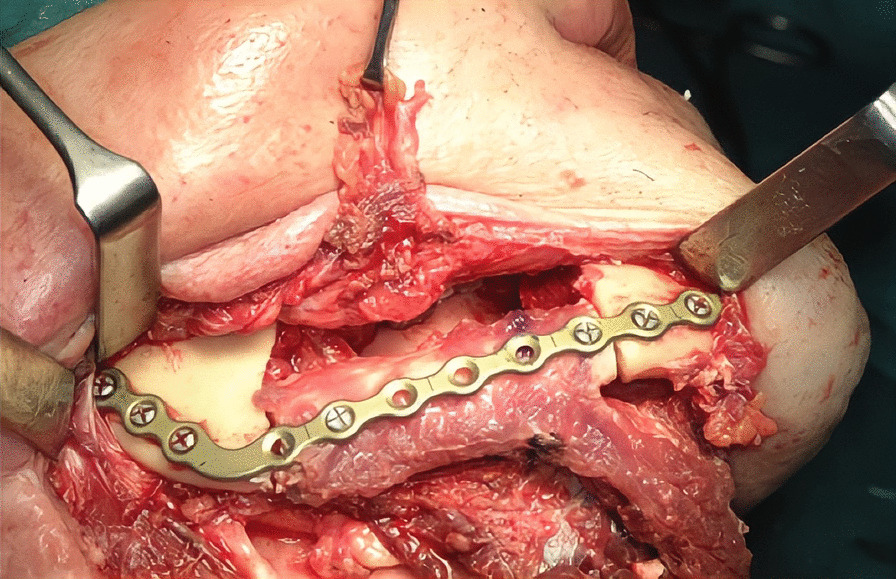
Intraoperative view after flap insetting, right side

**Fig. 10:**
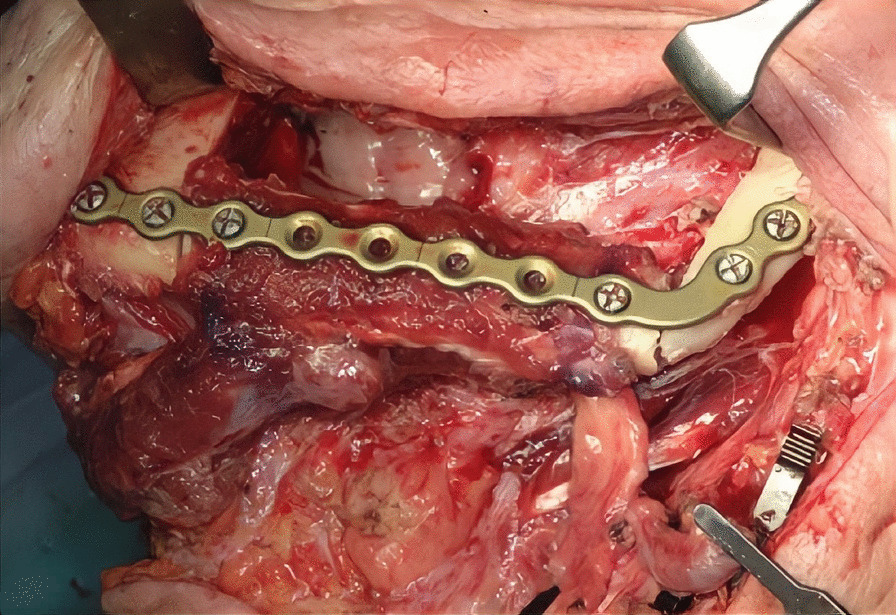
Intraoperative view after flap insetting, left side

**Fig. 11:**
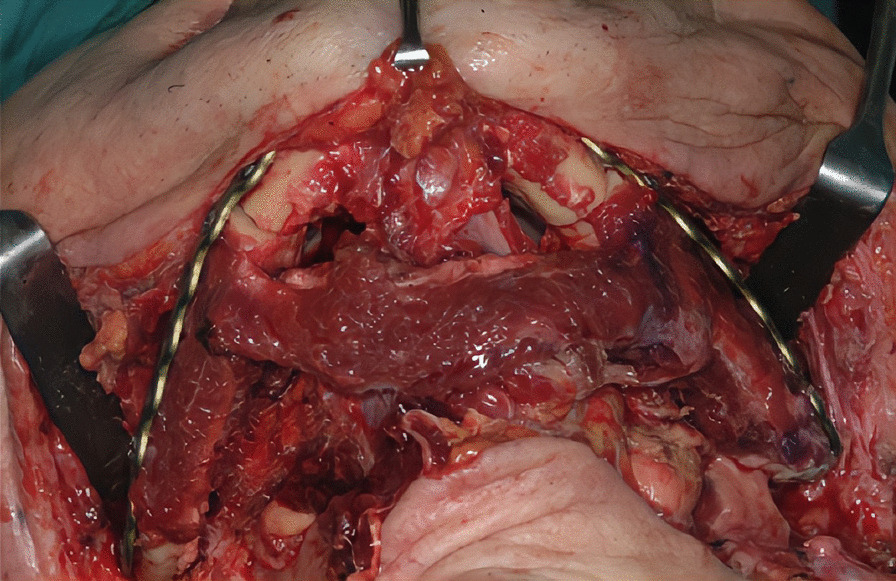
Intraoperative view after flap insetting, bottom-up perspective

**Fig. 12:**
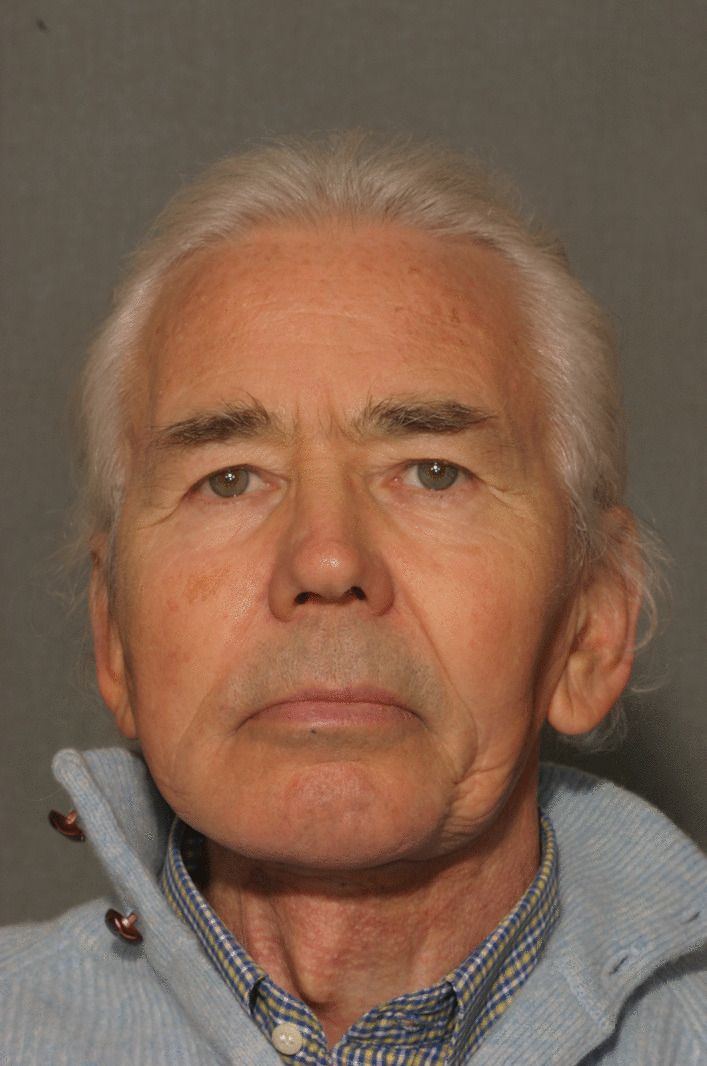
Postoperative frontal view of the patient

**Fig. 13:**
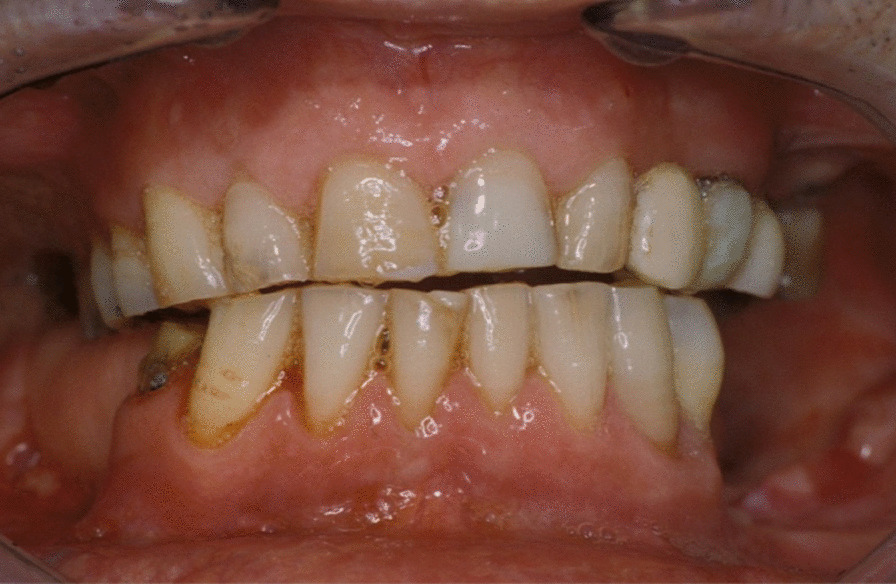
Postoperative intraoral view of the patient

**Fig. 14:**
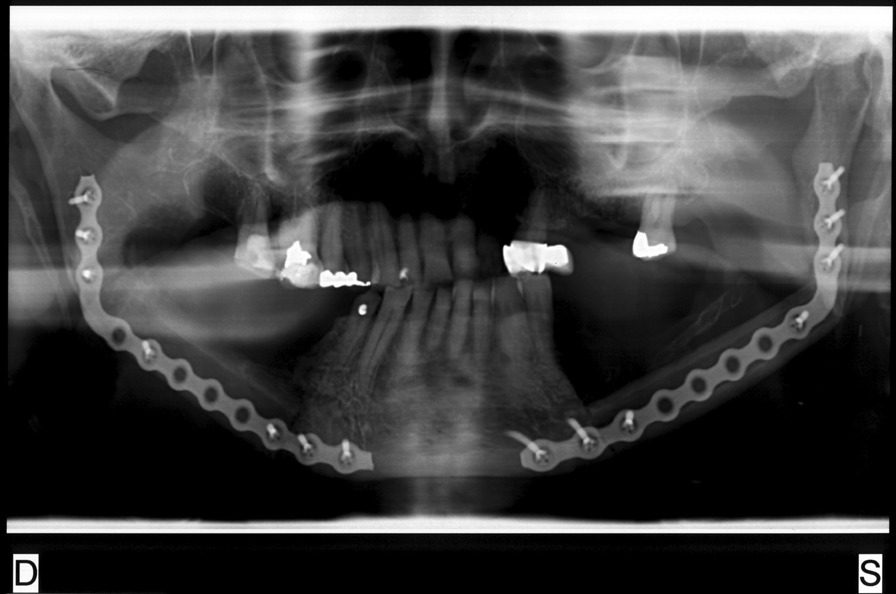
Postoperative orthopantomography

The first step was preplating which consisted of using a 2.0 mm reconstruction plate from the branch to the symphysis for symphyseal fixation on each and simultaneously using an intraoperative bite with a rigid intermaxillary block and intermaxillary fixation (IMF) screws to optimize the occlusal reference plane (Figs. 4, 5, 6). We then proceeded to carry out a bilateral segmental mandibulectomy from the angle to the mental foramen to resect both mandibular bodies. Particular attention was directed during this phase of the procedure to limiting as much as possible the dissection of the mandibular symphysis and the ascending branches in order to prevent devascularization since both inferior alveolar arteries would have been sacrificed. Contemporaneously with the surgical resection process, we proceeded to harvest one free fibular osteomuscular flap upon which the osteotomies planned were performed. We then proceeded to carry out a subperiosteal dissection of the central segment of the flap in order to remove the portion of the fibula that had become unnecessary since the symphysis had been preserved. The blood supply for tissue reconstruction was assured by the peroneal artery and accompanying veins wrapped in the periosteal layer (Figs. 7, 8). Despite our efforts to limit the dissection of the mandibular symphysis, we were still concerned about the long term vitality of that portion and the most distal portion of the reconstruction. In view of that consideration and the specific pecularities characterizing this particular case as well as the risks that are intrinsically linked to reconstructive surgery, we had prepared a secondary plan foreseeing recourse to a second flap starting from the contralateral fibula should there be any loss of vitality of one or both bone segments. Fortunately, no loss of vascularization was noted at any point, and after the free flap was inset the connecting pedicle reclined at the bridge caudally with respect to the preserved symphysis region (Figs. 9, 10, 11). An anastomosis was instead performed on the left side of the neck linking the pedicle of the flap with the external carotid artery, the lingual vein and the external jugular vein. The post-operative period passed without any relevant complications. Five years later at the time of the patient’s unexpected death, which was correlated to other causes, he showed good clinical and radiographic signs of bone consolidation (Figs. 12, 13, 14).

## Discussion

The involvement of both sides of the mandible is a serious but uncommon complication of radiation therapy for head and neck malignancies. In the management of this scenario, several factors must be taken into account, such as general conditions and health status of the patient, hard and soft tissue defect extent and vessel conditions. While conservative treatment such as the administration of antibiotic therapy, hyperbaric oxygen therapy or limited debriding may prove successful in a share of cases, a remarkable amount of patients requires more aggressive treatments due to recurrence of infections or to excruciating pain, as was the presented case. Concerning reconstructive strategies, ORN-related endothelial damage leads to poor oxygen diffusion and therefore may impair wound healing [9]. As previously mentioned, the use of vascularized bony tissues represents the best option to deal with such defects. Given the availability of bone tissue and the pedicle length, fibula flap is probably the most versatile flap to address similar scenarios; this can be accomplished in multiple ways. In fact, other therapeutic options could have been used in the case described. For example, each resected segment could have been replaced at that time or at a later date with a single vascularized flap [10]. It must nevertheless be remembered that patients with ORN generally present a poor vascular patrimony both because of the radiotherapy itself and the surgical procedures that they might have undergone in the past [11]. The native mandibular symphysis could have been sacrificed regardless of its non-involvement in the ORN process generating in that way an angle to angle defect permitting a more traditional management as far as a single flap reconstruction was concerned. In the case described here, the surgeons’ aim was that of using a technically more complex methodology that, if successful, could avoid recourse to more than one flap and/or to more than one procedure. At the same time, they intended to preserve the mandibular symphysis and relative dental elements to assure a good functional outcome as far as speech and swallowing were concerned by maintaining the attachment of the tongue and laryngeal muscles. Crucially, the labial competence and the projection of the soft tissues of the lower third of the face were preserved thus contributing to a satisfactory facial aesthetic which would no doubt have a positive impact on the patient’s psychological well-being and self-esteem. The technique adopted here permitted us to reach these aims limiting the procedure’s morbidity both immediately and over the long term. The underlying premise was based on the anatomic peculiarity of a vascularization of the fibula enabling the surgeon to perform osteotomies and consequent independent bone segments to be perfused by a single pedicle. The periosteal collaterals of the peroneal artery provide the blood supply of the single elements. As has been noted in the past and was demonstrated even in this case, removing bone segments a few centimeters long from a fibula flap does not compromise the blood supply of the flap when the dissection and preservation of the periosteal plane and relative muscle are carefully planned and executed. Jacobson et al. [12] first reported that the distal segment of the fibula could be vascularized by the periosteal vessels passing circumferentially around the fibula from the distal continuation of the peroneal artery and by perfusion through the connected periosteum. Fan et al. [13] reported a series of 31 patients who underwent bilateral reconstruction of advanced ORN in the mandible using a single fibular osteocutaneous flap; in 29 cases, the native symphysis was preserved, with an approach that can be considered substantially analogous to the case we described. Considering a follow-up time ranging from 7 to 72 months, all fibular flaps survived, with only a partial necrosis of the skin paddle reported in one case. As pointed out by the aforementioned Authors, this technique faces a drawback when the resection is extended to the ascending ramus because of the limitation of movements determined by the central periosteal bridge. To cope with such condition without harvesting a second flap, some Authors [3] proposed separating the central bridge and generate therefore two distinct flaps from a single fibula. However, this implies the need to prepare two recipient pedicles (which can be challenging in a vessel-depleted neck) and consequently two flap pedicles with an adequate length. Last, two separate skin paddles based on perforators should also be designed.

## Conclusion

To conclude, the technique described here using a single flap proved to be safe and efficacious in a case presenting multifocal osteonecrosis of the jaw. The morbidity of the procedure was limited because the tissue resection and reconstruction processes were minimized. In cases such as this one, the surgeon can choose the side of the neck that is most suitable for the anastomosis and that will facilitate a rapid post-surgical recovery even in a patient with vessel-depleted neck.

## Data Availability

Not applicable.
